# Application of a human lectin array to rapid in vitro screening of sugar-based epitopes that can be used as targeting tags for therapeutics

**DOI:** 10.1093/glycob/cwaf011

**Published:** 2025-03-02

**Authors:** Stefi V Benjamin, Maureen E Taylor, Kurt Drickamer

**Affiliations:** Department of Life Sciences, Sir Ernst Chain Building, Imperial College, London SW7 2AZ, United Kingdom; Department of Life Sciences, Sir Ernst Chain Building, Imperial College, London SW7 2AZ, United Kingdom; Department of Life Sciences, Sir Ernst Chain Building, Imperial College, London SW7 2AZ, United Kingdom

**Keywords:** carbohydrate-binding proteins, glycomimetics, glycotherapeutics, lectins, oligosaccharides

## Abstract

An increasing number of clinical applications employ oligosaccharides as tags to direct therapeutic proteins and RNA molecules to specific target cells. Current applications are focused on endocytic receptors that result in cellular uptake, but additional applications of sugar-based targeting in signaling and protein degradation are emerging. These approaches all require development of ligands that bind selectively to specific sugar-binding receptors, known as lectins. In the work reported here, a human lectin array has been employed as a predictor of targeting selectivity of different oligosaccharide ligands and as a rapid in vitro screen to identify candidate targeting ligands. The approach has been validated with existing targeting ligands, such as a synthetic glycomimetic GalNAc cluster ligand that targets siRNA molecules to hepatocytes through the asialoglycoprotein receptor. Additional small oligosaccharides that could selectively target other classes of cells have also been identified and the potential of larger glycans derived from glycoproteins has been investigated. In initial screens, potential ligands for targeting either vascular or sinusoidal endothelial cells and plasmacytoid dendritic cells have been identified. Lectin array screening has also been used to characterize the selectivity of glycolipid-containing liposomes that are used as carriers for targeted delivery. The availability of a rapid in vitro screening approach to characterizing natural oligosaccharides and glycomimetic compounds has the potential to facilitate selection of appropriate targeting tags before undertaking more complex in vivo studies such as measuring clearance in animals.

## Introduction

Use of oligosaccharides (glycans) to target proteins, lipids and RNA molecules to specific tissues and cells has become a viable clinical approach relatively recently. Glycan targeting is mediated by various glycan-binding receptors (lectins) on the surfaces of specific cell types. For example, in enzyme replacement therapy for lysosomal storage disorders, enzymes are tagged with sugars that direct them to the mannose receptor on macrophages or to the mannose 6-phosphate receptor found on a wide range of cells (Grabowski et al. 1995; Lee et al. 2003). More recently, a new class of siRNA treatments to reduce expression of blood proteins produced in the liver have been directed to the asialoglycoprotein receptor on hepatocytes with a glycan tag (Nair et al. 2014; Brannagan 3rd et al. 2022). The tag used in these cases is a cluster of GalNAc residues identified through a series of ligand-optimization studies (Prakash et al. 2016).

Targeting liposomes through glycan tags has been investigated in vitro using GM1, GM3 and synthetic sialoside ligands that interact with sialoadhesin (siglec-1/CD169) or other siglecs (Affandi et al. 2020; Shen et al. 2024) and there have been some early-stage clinical applications (Arena et al. 2022). In addition to these applications that have reached the clinic, other potential targeting strategies using glycan have been proposed. For example, antibodies tagged with sugar epitopes, known as lysosome-targeting chimeras (LYTACs) can potentially clear specific proteins from cell surfaces and from circulation (Banik et al. 2020; Ahn et al. 2021; Wang C et al. 2024. Reshaping the tumor microenvironment by degrading glycoimmune checkpoints Siglec-7 and -9. bioRxiv. https://doi.org/10.1101/2024.10.11.617879). In all of these cases, successful targeting depends on selective binding of the glycan tag to an appropriate receptor and significant in vivo screening, including extensive clearance studies in animals, is required in the design of appropriate glycan conjugates.

Mammalian lectin arrays have recently been developed for rapid screening of glycoconjugate binding to a panel of sugar-binding receptors (Jégouzo et al. 2020; Benjamin et al. 2024). These arrays can be used to test binding of glycans on bacteria, viruses, fungi and parasites in order to investigate how these micro-organisms interact with cells in the innate immune system. In addition to demonstrating the roles of these receptor in pathogen recognition, availability of a comprehensive panel of human sugar-binding receptors could also facilitate rapid in vitro screening of glycans and glycomimetics to assess their potential for in vivo targeting.

The utility of the human lectin array for characterizing the interactions of existing targeting glycans has now been demonstrated and additional oligosaccharides that can potentially be used as receptor- and cell-specific delivery tags have been identified.

## Results

### Strategy

**Figure 1:**
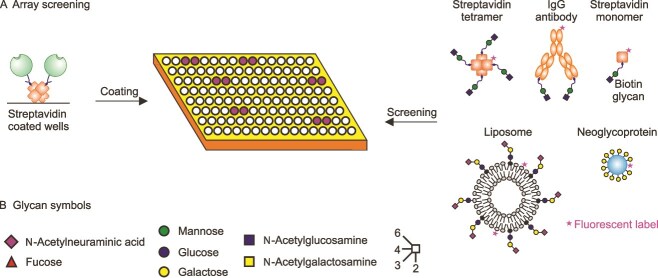
Strategy for screening of lectin array with receptor-selective glycan epitopes. A) Methods for presentation of glycans as multivalent, tetravalent, bivalent and monovalent ligands are summarized. B) Sugar symbols used here and in subsequent figures. Unless otherwise indicated, linkages from galactose and GlcNAc residues are in β configuration and linkages from NeuAc, fucose, mannose, and GalNAc are in α configuration.

The current version of the human lectin array displays 39 different carbohydrate-recognition domains (CRDs) from 36 receptors, covering 7 different structural categories of lectins (Table 1). The use of biotinylation tags to tether the CRDs to streptavidin-coated wells ensures that the binding sites are project away from the surface of the wells and avoids potential chemical modifications near to the binding site that might occur with chemical derivatization (Benjamin et al. 2024). Several approaches were used to test the ability of oligosaccharide epitopes to target receptors selectively (Fig. 1). The primary strategy was to use biotinylated glycans complexed with fluorescently-labelled streptavidin for initial screening. Screening was repeated at a 25- to 100-fold range of concentrations. In most cases, the results were similar across concentrations, suggesting that binding is relatively high affinity, but in a few cases binding to some receptors decreases with reduced ligand concentration, consistent with weaker binding.

**Table 1:** CRDs displayed on human lectin array.

Abbreviation	Protein	Gene
MBP	Mannose-binding protein/lectin	MBL2
SP-A	Surfactant protein A	SFTPA1
SP-D	Surfactant protein D	SFTPD
ColK1	Collectin K1	COLEC11
MMR CRD 4	Mannose receptor/CD206 C-type CRD 4	MRC1
Langerin	Langerin	CD207
DC-SIGN	DC-SIGN/CD209	CD209
DC-SIGNR	DC-SIGNR/L-SIGN/CD299	CLEC4M
Prolectin	Prolectin	CLEC17A
LSECtin	LSECtin	CLEC4G
Endo180 CRD 2	Endo180/UPARAP	MRC2
Mincle	Mincle	CLEC4E
Dectin-2	Dectin-2	CLEC6A
BDCA-2	Blood dendritic cell antigen 2	CLEC4C
Dectin-1	Dectin-1	CLEC7A
ASGPR1	Asialoglycoprotein receptor subunit 1	ASGR1
ASGPR2	Asialoglycoprotein receptor subunit 2	ASGR2
MGL	Macrophage galactose receptor	CLEC10A
SRCL	Scavenger receptor C-type lectin	COLEC12
Galectin-1	Galectin 1	LGALS1
Galectin-2	Galectin 2	LGALS2
Galectin-3	Galectin 3	LGALS3
Galectin-7	Galectin 7	LGALS7
Galectin-4 N	Galectin 4 N-terminal CRD	LGALS4
Galectin-4C	Galectin 4 C-terminal CRD	LGALS4
Galectin-8 N	Galectin 8 N-terminal CRD	LGALS8
Galectin-8C	Galectin 8 C-terminal CRD	LGALS8
Galectin-9 N	Galectin 9 N-terminal CRD	LGALS9
Galectin-9C	Galectin 9 C-terminal CRD	LGALS9
Siglec-1	Sialoadhesin	SIGLEC1
Siglec-3	CD33	CD33
Siglec-5	Siglec 5	SIGLEC5
Siglec-7	Siglec 7	SIGLEC7
Siglec-9	Siglec 9	SIGLEC9
Siglec-11	Siglec 11	SIGLEC11
Intelectin-1	Intelectin 1	ITLN1
Intelectin-2	Intelectin 2	ITLN2
MMR-R	Mannose receptor R-type CRD	MRC1
Ficolin 1	Ficolin 1/Ficolin M	FCN1
Chl3-L2	Chitinase 3-like lectin 2/YKL39	CHI3L2

Ligands that showed selective binding in the tetravalent format could then be further tested with streptavidin modified so that it forms only monomers. In some cases, bivalent complexes were formed with antibodies to biotin and highly multivalent ligands were either serum albumin with covalently attached sugars or liposomes. Comparison of these results provides some insights into whether selective binding results from affinity for a single oligosaccharide or requires multivalent presentation.

### Monosaccharides versus oligosaccharides

**Figure 2:**
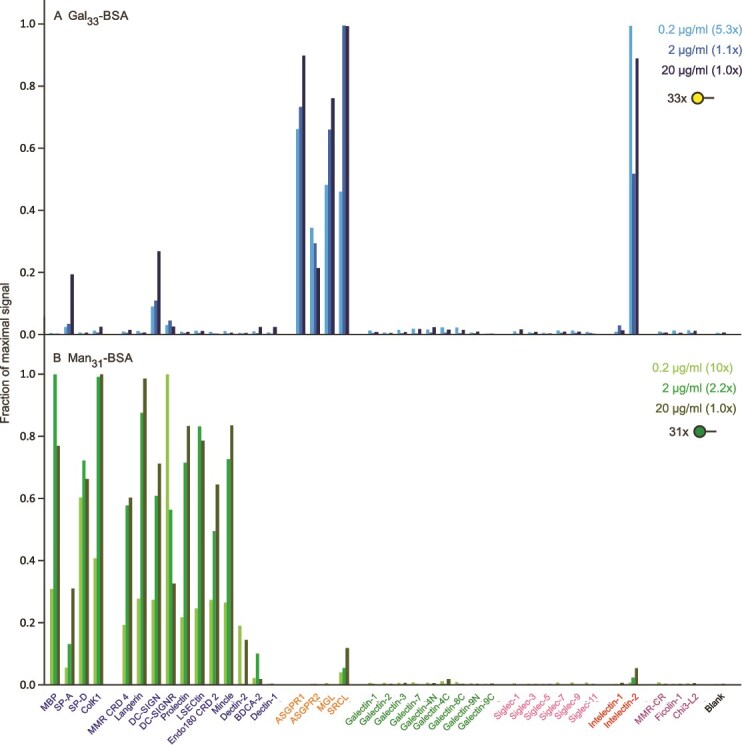
Screening of lectin array with FITC-labelled neoglycoproteins. A) Gal_33_-BSA. Errors ranged from 8%–13%. B) Man_31_-BSA. Errors ranged from 13%–25%. Full data are presented in Table S1. In this and subsequent figures, the expansion needed to make the highest peak for the more dilute concentrations of ligands equal to the highest peak at the highest concentration for that ligand is indicated next to each concentration. Because the normalized values are affected by different affinities of ligands for the different receptors, some ligand-receptor pairs give a smaller relative signal as the concentration of ligand increases. Such results appear counter-intuitive, but in all such cases the absolute fluorescence increases with increasing ligand concentration.

Screening the array with simple monosaccharide ligands, such as biotinylated galactose, complexed with streptavidin failed to show consistent binding to any receptors. In contrast, screening with the highly multivalent neoglycoprotein ligands Man_31_-BSA and Gal_33_-BSA in each case showed binding to multiple CRDs (Fig. 2), confirming that the monosaccharides bind, but must be highly multivalent to achieve sufficient avidity to withstand washing of the array. While the binding shows clear selectivity of mannose and galactose for different classes of CRDs, it also demonstrates that effective targeting requires more complex oligosaccharide ligands. These results correlate with the finding that neoglycoproteins made with monosaccharides often target multiple tissues (Schlesinger et al. 1980).

**Figure 3:**
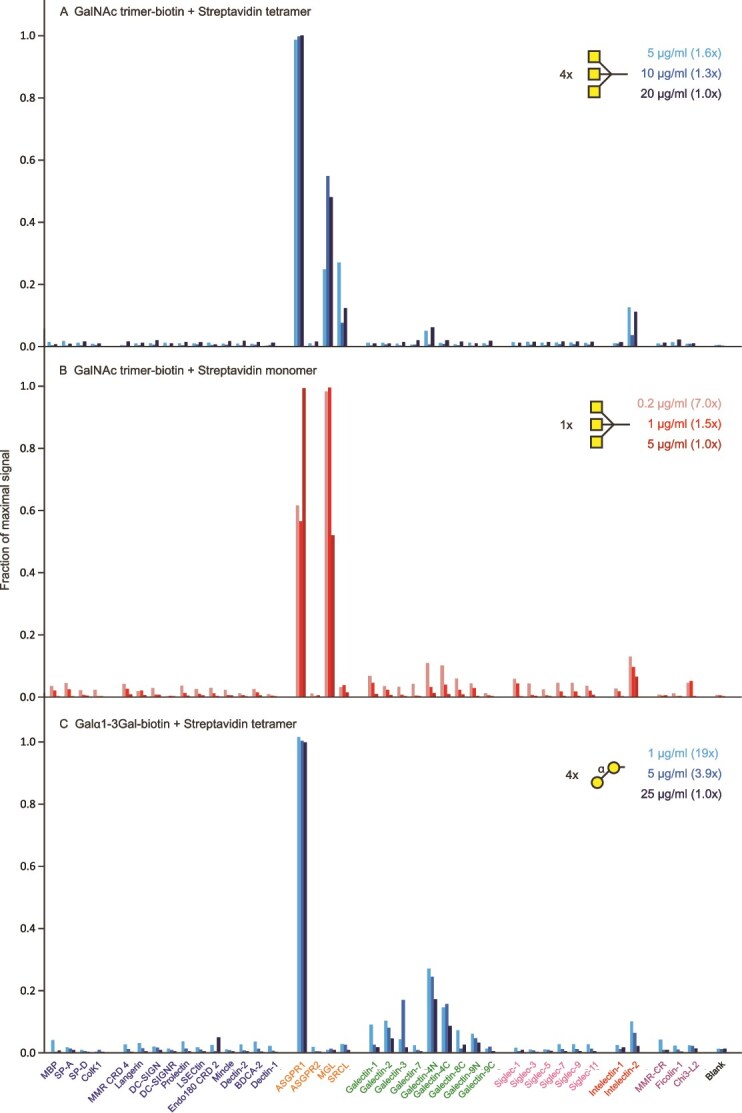
Screening of lectin array with GalNAc_3_ cluster and Galα1–3Gal ligands. A) GalNAc_3_ cluster ligand complexed with streptavidin tetramer. Errors ranged from 11–28%. B) GalNAc_3_ cluster ligand complexed with streptavidin monomer. Errors ranged from 2%–16%. C) Galα1–3Gal ligand complexed with streptavidin tetramer. Errors ranged from 9%–24%. Full data are presented in Tables S2 and S3.

A widely used targeting glycomimetic developed for directing siRNA molecules to hepatocytes through the asialoglycoprotein receptor contains a cluster of three GalNAc residues (Prakash et al. 2016; Brannagan 3rd et al. 2022). Streptavidin tetramers complexed with a biotinylated form of this synthetic cluster ligand showed very selective binding, with the strongest signals observed for the major subunit of the asialoglycoprotein receptor over a range of concentrations (Fig. 3A). However, the results also indicate that this ligand binds to the macrophage galactose receptor. A similar pattern of binding was observed for a monomeric version of this ligand (Fig. 3B), reflecting tight binding of the ligand through a high-affinity binding site. Interestingly, a Galα1–3Galβ1–4GlcNAc trisaccharide complexed with streptavidin shows high selectivity for the asialoglycoprotein receptor without binding the macrophage receptor, although it does interact weakly with some of the galectins (Fig. 3C). However, this oligosaccharide does not show selective binding as a bivalent antibody complex (Table S3).

The array results with the GalNAc cluster ligand correlate with observations in mice showing hepatic accumulation of siRNA conjugated to this ligand, which has been optimized for targeting the asialoglycoprotein receptor with a single glycan attached to one end of an RNA molecule (Prakash et al. 2016; Brannagan 3rd et al. 2022). These experiments demonstrate the utility of the lectin array for demonstrating selective binding in a simple in vitro experiment that correlates with animal studies. The observed binding to the macrophage galactose receptor also shows that binding to additional receptors may not always be detected in animal clearance studies, in this case probably because of the relative abundance of the hepatic receptor. A key point about the array screening results is that results for receptors that show fluorescence at the level of the blank are informative, since the absence of binding to non-target receptors demonstrates selectivity.

### Identification of additional glycan epitopes with narrow receptor selectivity

Screening with naturally occurring di- and trisaccharides revealed several additional candidates for selective receptor targeting. The targeting potential of these ligands depends both on a narrow receptor-binding profile for the glycan, often suggested by glycan array screening (functionalglycomics.org), and on restricted expression of the receptor on specific cell types. Glycans that meet these criteria include the disaccharide GlcNAcβ1–2Man, which interacts almost exclusively with LSECtin, a receptor found only on sinusoidal endothelial cells (Powlesland et al. 2008; Liu et al. 2004), and the Lewis^x^ trisaccharide, which binds particularly well to the scavenger receptor C-type lectin (SRCL) that is found more generally on endothelial cells (Coombs et al. 2005; Ohtani et al. 2001; Graham et al. 2011).

**Figure 4:**
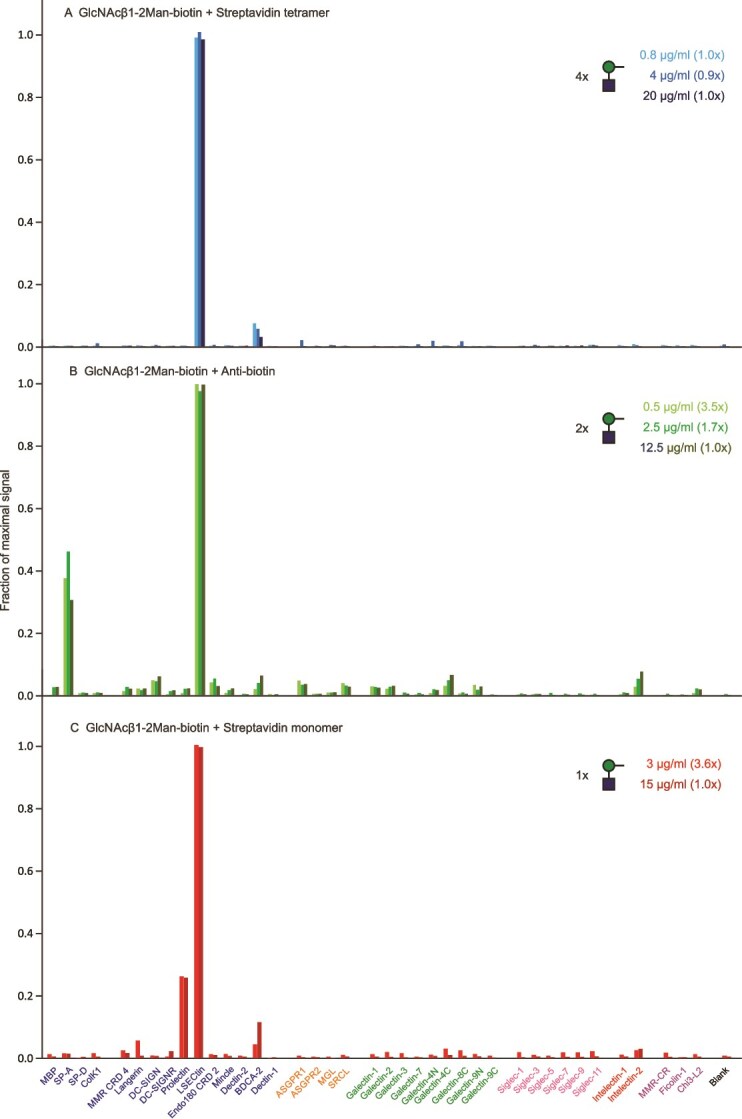
Screening of lectin array with GlcNAcβ1–2-man disaccharide. A) Complex with streptavidin tetramer. Errors ranged from 2%–27%. B) Complex with anti-biotin antibody. Errors ranged from 1%–5%. C) Complex with streptavidin monomer. Errors ranged from 12%–18%. Full data are presented in Table S4.

Binding of GlcNAcβ1–2-Man to LSECtin is highly selective even as the valency is reduced from tetrameric streptavidin complexes (Fig. 4A) to the bivalent antibody complexes (Fig. 4B). Binding to surfactant protein SP-A was observed for all antibody complexes tested and reflects the interaction of the CRD from SP-A to the antibody Fc region in a carbohydrate-independent manner (Lin and Wright 2006). Binding also remains largely selective with a monomeric complex (Fig. 4C), although some binding to the B cell-specific receptor prolectin is observed.

**Figure 5:**
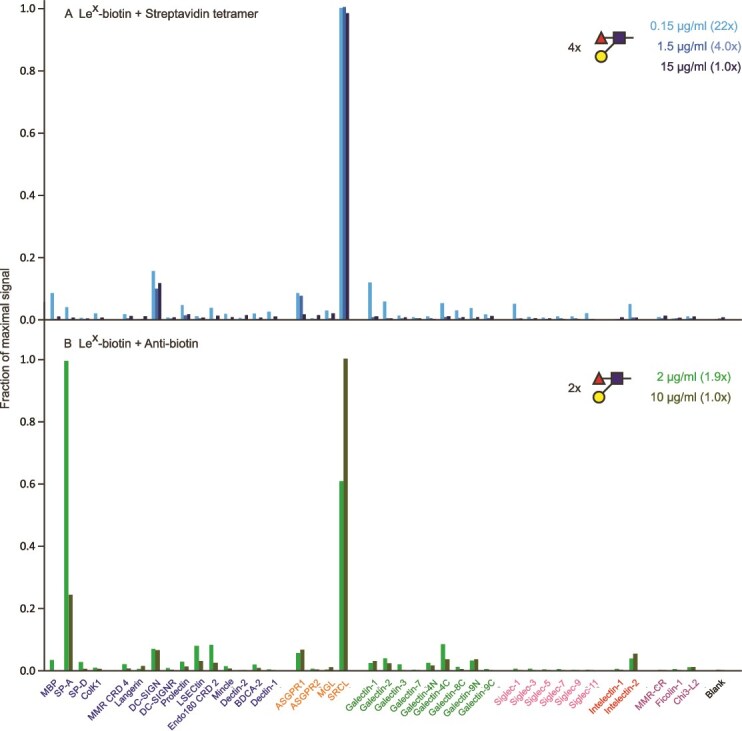
Screening of lectin array with Galβ1–3(Fucα1–4)GlcNAc (Le^X^) trisaccharide. A) Complex with streptavidin tetramer. Errors ranged from 4%–24%. B) Complex with anti-biotin antibodies. Errors ranged from 6–9%. Full data are presented in Table S5.

Binding of the Le^x^ trisaccharide Galβ1–3(Fucα1–4)GlcNAc shows high selectivity for SRCL both as a tetrameric complex (Fig. 5A) and as a bivalent antibody complex (Fig. 5B). However, very little binding of a complex with streptavidin monomers was observed and it was not selective for SRCL (Table S5). Thus, the results for these two simple oligosaccharides suggest that selective binding to endothelial clearance receptors could be achieved with dimeric presentation of sugar epitopes.

**Figure 6:**
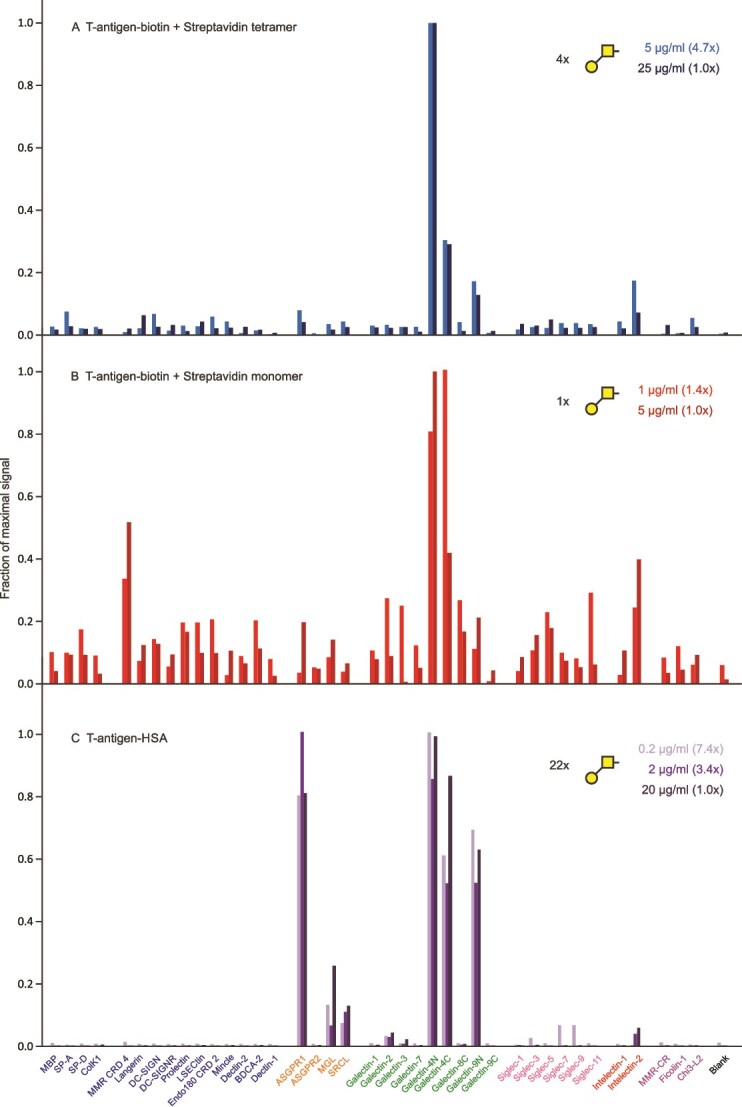
Screening of lectin array with Galβ1–3GalNAc-α- (T-antigen) disaccharide. A) Complex of oligosaccharide with streptavidin tetramer. Errors ranged from 7%–28%. B) Complex of oligosaccharide with streptavidin monomer. Errors ranged from 17%–30%. C) FITC-labelled T-antigen-human serum albumin (HSA) neoglycoprotein. Errors ranged from 6%–9%. Full data are presented in Table S6.

A tetrameric complex of the T-antigen disaccharide Galβ1–3GalNAc-α- largely targets galectin-4 (Fig. 6A), through both the N- and C-terminal CRDs and preferential binding to galectin-4 is also evident in a monomeric complex (Fig. 6B). In this case, a multivalent neoglycoprotein was also available and showed binding to galectin-4, but also binding to the N-terminal CRD of galectin-9 and to the asialoglycoprotein receptor (Fig. 6C). Galectin-4 is expressed almost exclusively in the gut, so in this environment the interaction with Galβ1–3GalNAc would likely be largely selective. Given the involvement of galectin-4 in tumor metastasis in the digestive system, the result suggests that inhibitory oligosaccharides based on the T-antigen could selectively block key interactions (Huflejt and Leffler 2004).

### Screening with natural glycans

**Figure 7:**
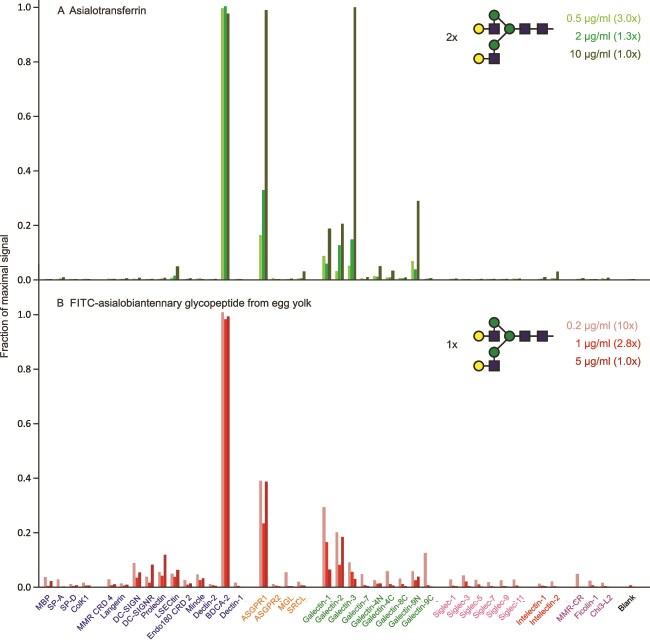
Screening of lectin array with Galβ1–4GlcNAc (LacNAc) disaccharide-containing conjugates of complex N-linked glycans. A) FITC-labeled asialotransferrin. Ranges for errors ranged from 4%–25%. B) FITC-labelled egg oligosaccharide. Errors ranged from 10%–22%. Full data are presented in Table S7.

In addition to small oligosaccharides, some larger N-linked glycans also show significant selectivity for receptors. The two N-glycosylation sites in human transferrin are predominantly occupied by biantennary, complex glycans, with a very small amount of more branched structures (Yamashita et al. 1989). Screening the array with desialylated transferrin showed binding to the dendritic cell antigen-2 (BDCA-2), a receptor that is expressed almost exclusively on plasmacytoid dendritic cells (Fig. 7A) (Dzionek et al. 2001). This binding is consistent with the binding characteristics of BDCA-2, which interacts predominantly with exposed Galβ1–4GlcNAc termini of these glycans (Jégouzo et al. 2015). Binding to other CRDs on the array, including the asialoglycoprotein receptor and some of the galectins, is very concentration-dependent, suggesting that binding to these CRDs reflects lower affinity interactions.

In order to confirm that binding to BDCA-2 results from the presence of biantennary glycans on asialotransferrin, an equivalent glycan generated from chicken egg yolk glycopeptide was directly labelled with FITC and used to screen the array (Fig. 7B). The binding profile largely mirrors what was observed for asialo-transferrin. The low level of binding to several CRDs such as DC-SIGN, DC-SIGNR, prolectin, LSECtin and others, may result from the presence of a small fraction of glycans lacking a terminal galactose residue on one branch (Liu et al. 2017), thus leaving an exposed terminal GlcNAc residue that can bind to these CRDs. Although the N-linked glycans in transferrin are closely spaced and might both interact with immobilized CRDs, binding of the monomeric glycan suggests that such multivalent interaction is not necessary to achieve selectivity. The very cell-type specific expression of BDCA-2 and the role of blood dendritic cells in regulating production of interferon α make this a potentially useful targeting interaction (Dzionek et al. 2001).

**Figure 8:**
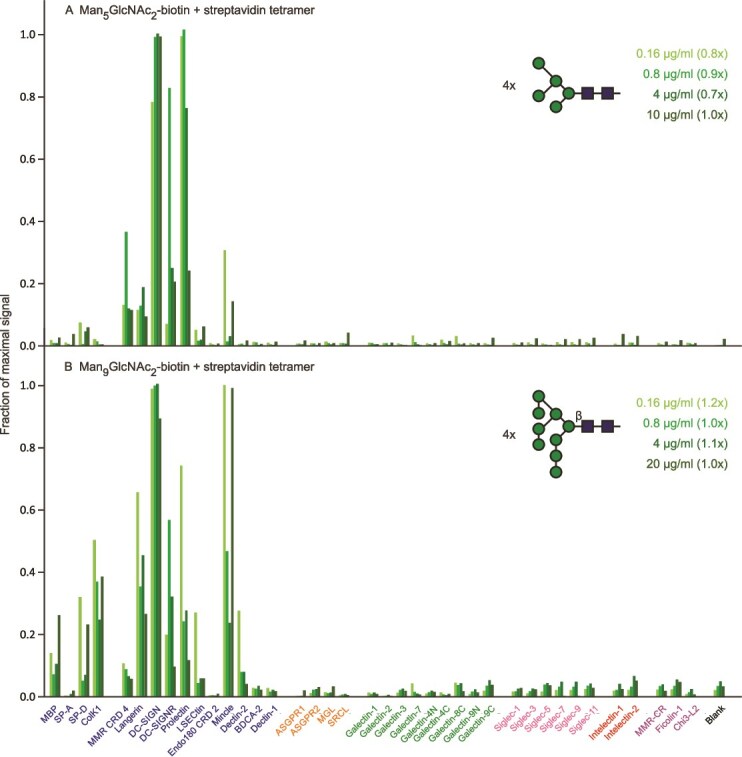
Screening of lectin array with oligomannose-containing glycan ligands. A) Man_5_GlcNAc_2_. Errors ranged from 4%–15%. B) Man_9_GlcNAc_2_. Errors ranged from 11%–17%. Full data are presented in Table S8.

In contrast to very selective binding of the complex N-linked glycans, oligomannose-type N-linked glycans interact with a wide range of CRDs (Fig. 8). Both Man_5_- and Man_9_-containing glycans bind most effectively to the dendritic cell receptor DC-SIGN, but both also bind to multiple other receptors. Thus, while these oligosaccharides have shown some potential for targeting to dendritic cells through DC-SIGN (Valverde et al. 2020), it may prove difficult to make this receptor a target when all of the other potential targets are considered.

The Man_5_ glycan is somewhat more selective than Man_9_, particularly because it shows a much reduced signal for CRDs from the soluble serum collectins. The results suggest that the terminal Manα1–2Man caps on the Man_9_ glycan may be responsible for the additional interactions, although screening with short Manα1–2Man polymers on their own did not demonstrate selective targeting (Table S8).

### Screening with liposomes

**Figure 9:**
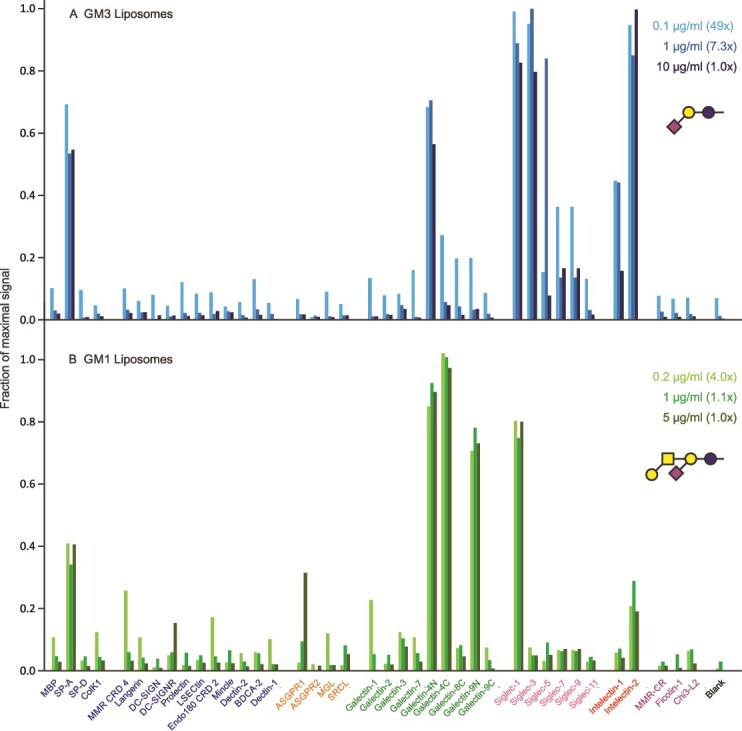
Screening of lectin array with ganglioside-containing liposomes. Liposome concentrations are indicated in μg/mL of total lipid. A) GM1. Errors ranged from 7%–14%. B) GM3. Errors ranged from 14%–33%. Full data are presented in Table S9.

Liposomes containing either GM1 or GM3 gangliosides have been used to target the sialic acid-binding receptor sialoadhesin on macrophages (Siglec-1; CD169) (Affandi et al. 2020; Shen et al. 2024). Screening of the lectin array with fluorescently labelled liposomes containing these gangliosides confirmed that both show strong binding to sialoadhesin (Fig. 9). Amongst the siglecs tested, GM1-containing liposomes are selective for sialoadhesin, while those containing GM3 interact with siglec 3 (CD33) to a similar extent and more weakly with other siglecs. Thus, in terms of targeting siglecs, GM1-containing liposomes are potentially more selective. Some of the interactions observed, such as the binding of GM3-containing liposomes to intelectin 1, have not been reported in glycan array screening (Wesener et al. 2015), but our screening of the Consortium for Functional Glycomics array with intelectin 1 suggests weak interaction with the GM3 headgroup (unpublished data). The highly multivalent presentation in liposomes may enhance such weak binding and thus reduce selectivity of targeting.

Both types of liposomes interact with the N-terminal CRD from galectin-4, but the GM1 liposomes also bind to the C-terminal CRD of galectin 4 and the N-terminal CRD of galectin 9. This binding pattern is reminiscent of the binding of T-antigen to these same galectin CRDs (Fig. 6), reflecting the presence of the Galβ1–3GalNAc structure in the headgroup of GM1. GM1 also binds to intelectin-2, probably through the galactose residue (unpublished observations). Binding to the CRD from pulmonary surfactant protein SP-A is observed for all liposome preparations tested, including those lacking glycans, and thus likely reflects the well characterized capacity of this CRD to bind lipids (McCormack et al. 1997). Given the distinct sites of expression of the siglecs, largely in the immune system, and galectins 4 and 9 as well as intelectin-2 in the gut and other epithelia, targeting of injected liposomes would presumably be primarily dictated by the different siglec-binding properties of the gangliosides (Huflejt and Leffler 2004; Nonnecke et al. 2022; Gonzalez-Gil and Schnaar 2021).

## Discussion

Table 2 summarizes the binding interactions reported here, along with information about the localization of some of the human glycan-binding receptors that can be selectively targeted by oligosaccharide ligands. The results indicate that this screening can provide useful guidance for development of sugar-based targeting tags. A key outcome is identification of some simple oligosaccharides that show strong selectivity for receptors that have restricted tissue distributions. The sugar structures tested were chosen because they have been shown in previous glycan array studies to bind one or more of the receptors. However, a key point about the array screening results is the absence of binding to other receptors, since the absence of binding to non-target receptors is needed to demonstrate selectivity. Glycan array results do not readily provide a direct comparison of binding of different receptors to the same ligands.

**Table 2:** Summary of selective ligand-receptor interactions. Valency indicates the lowest valency of ligand that shows selective binding.

Ligand	Receptors	Cells/Tissues	Valency
GalNAc_3_	ASGPR >	Hepatocytes	Monovalent
	MGL	Macrophages	
GlcNAcβ1–2Man	LSECtin	Sinusoidal endothelial cells: liver/lymph nodes	Dimeric
Galβ1–4(Fucα1–3)GlcNAc	SRCL	Endothelial cells	Dimeric
Lewis^x^			
Galβ1–3GalNAcα1-Ser	Galectin-4	Intestine	Polyvalent
T-antigen			
Gal_2_GlcNAc_2_Man_3_GlcNAc_2_	BDCA-2	Blood dendritic cells	Dimeric
Biantennary glycan-biotin	ASGPR	Hepatocytes	Monomeric
NeuAc2–3(Gal1–3GalNAc1–4)Gal1–4Glc	Sialoadhesin >	Macrophages	Polyvalent
		Intestine	
Ganglioside GM1	Galectin 4/8 + Intelectin 2		
NeuAc2–3Gal1–4Glc	Sialoadhesin + Siglec 3/5 >	Macrophages	Polyvalent
Ganglioside GM3	Galectin 4 + Intelectins 1/2	Intestine	
Man_5_GlcNAc_2_	DC-SIGN	Dendritic cells	Tetravalent
	Prolectin	B cells	

The results for the sugars tested here suggest that it may be possible to identify additional oligosaccharides with unique targeting potential. One potential candidate is GalNAc-4-SO_4_, which interacts with the R-type CRD in the macrophage mannose receptor (Fiete et al. 1998). Looking beyond mammalian glycans, microbial glycans may provide inspiration for other useful sugar epitopes. For example, although the mammalian mannose-terminated glycans show a disappointing lack of selectivity, substructures found in yeast mannan, such as Manα1–2Man oligomers, might show greater selectivity for receptors such as dectin-2 (Feinberg et al. 2017). Similarly, GlcNAc-containing polymers such as poly-N-acetylglucosamine (GlcNAcβ1–4GlcNAc) found in biofilms shows more restricted binding (Benjamin et al. 2024), suggesting other types of oligosaccharides that might be tested.

Successful development of the synthetic GalNAc cluster ligand demonstrates that improvements in affinity and cost can be achieved using glycomimetics (Prakash et al. 2016). Such synthetic analogs can be optimized by monitoring affinity for a proposed target receptor, but monitoring selectivity using the array yields complementary information, by providing a rapid in vitro method for screening out ligands that are less selective for the target. Obtaining this information in an in vitro assay could reduce the need for more complex studies in animals during the initial screening steps.

Screening the array can also flag up issues that may reduce targeting selectivity in vivo. The results confirm selective targeting by the GalNAc cluster ligand that is already employed in several approved clinical treatments that direct siRNA molecules to hepatocytes. Although the data suggest that some of this ligand will be directed to macrophages, the high capacity of the hepatic clearance system would mean that most of the siRNA would be directed to hepatocytes (Nair et al. 2014). The Galα1–3Galβ1–4GlcNAc trisaccharide shows higher selectivity for the asialoglycoprotein receptor without binding the macrophage receptor, but does not work as a monomeric ligand.

The results for desialylated egg glycopeptide suggest that even limited heterogeneity of natural glycans can results in some mis-targeting, although the resulting loss of selectivity could be prevented with appropriate quality control. The liposome screening experiments also demonstrate that in addition to binding to sialoadhesin, the intended target ligand on macrophages, natural gangliosides will likely also target both other siglecs and lectins in other families. Development of glycomimetics selective for individual siglecs addresses the first point (Nycholat et al. 2019), but screening against the larger complement of CRDs on the array would potentially provide further evidence of selectivity.

The results presented here are largely focused on oligosaccharide tags that target receptors for uptake into cells. However, receptor-selective oligosaccharides identified using the array can be employed in other applications, such as triggering signaling through receptors that activate intracellular kinases and phosphatases. Combining the selectivity results from the array with other information about receptor geometry can provide a basis for designing novel stimulatory ligands. For example, the demonstration that bi-antennary, galactose-terminated glycans bind preferentially to the BDCA-2 receptor on plasmacytoid dendritic cells, combined with structural information about the arrangement of BDCA-2 dimers, suggests ways to create ligands that could modulate interferon α production by forming BDCA-2 clusters (Liu et al. 2024).

## Materials and methods

### Glycoproteins

For fluorescein labelling of neoglycoproteins, 1 mg of Gal_33_-BSA or 1 mg of Man_31_-BSA from E-Y laboratories, or 250 μg of T-antigen_22_-human serum albumin from Dextra Laboratories was reacted with 12.5 μg FITC in 250 μL of 100 mM bicine, pH 9.0 for 2 h at room temperature. Excess reagent was removed by repeated washing with Tris-buffered saline in a VivaSpin-2 centrifugal concentrator with a 10-kDa cut-off membrane (VIVAproducts). Human transferrin (1 mg) from Sigma was digested with 1250 units of *Clostridium perfringens* neuraminidase from New England Biolabs in 50 μL of 50 mM sodium acetate buffer, pH 5.5, 5 mM CaCl_2_, for 20 h at 37 °C. The sample was diluted into 350 mM bicine, pH 9.0 and labelled as described above.

### Egg glycopeptide

Egg glycopeptide, prepared and desialylated as described previously (Jégouzo et al. 2015), was labelled with FITC and purified on a 1 × 30 cm BioGel P2 column run in Tris-buffered saline.

### Liposomes

Liposomes were prepared by combining 3 μmole of distearoyl phosphatidylcholine, 1.25 μmole of GM1 or GM3 gangliosides (Avanti Polar Lipids), 1.75 μmole of cholesterol, and 0.25 μg of aminofluorescein coupled to distearoyl phosphatidyl ethanolamine-polyethylene glycol 2000-N-hydroxysuccinimide (Cayman Chemicals). The mixture was dried, resuspended in 2 mL of TBS, sonicated for 1 min and extruded 5 times through 0.2 μm aluminum filters (Anitop).

### Complexes with biotinylated oligosaccharides

Biotinylated oligosaccharides GlcNAcβ1–2Man and Le^x^ were from Dextra Laboratories and T-antigen, Man_5_GlcNAc_2_ and GalNAc_3_ cluster ligand were from Sussex Research Laboratory. 1,2-α-1,2-α-D-mannotriose-1-O-ethylamine from Biosynth was reacted with biotinamidohexanoic acid *N*-hydroxysulfosuccinimide ester from Sigma. The product was purified by chromatography on BioGel P2 and Dowex-1 columns and characterized by mass spectrometry. AlexaFluor488-labelled streptavidin was from Life Technologies and AlexaFluor488-labelled anti-biotin was from Jackson Immunoresearch. Monomeric streptavidin from Sigma was labelled with FITC as described above. Biotinylated oligosaccharides were combined with streptavidin or antibody in a 10-fold molar excess compared to biotin-binding sites. Complexes were formed by incubation for 3 h at room temperature in Tris-buffered saline, pH 7.4, and were purified on a 15-mL column of Sephadex G-25 eluted with Tris-buffered saline.

### Array screening

All procedures were conducted in binding buffer containing 0.15 M NaCl, 25 mM Tris-Cl, pH 7.8, 2.5 mM CaCl_2_ in streptavidin-coated 96-well black plates (Life Technologies). Coating of wells with biotinylated CRDs was performed as previously described (Benjamin et al. 2024). Ligands diluted in binding buffer containing 0.1% bovine serum albumin were added in 60 μL aliquots. For complexes of biotinylated ligands, binding was performed in the presence of 20 mM free biotin. After incubation for 2–4 h at 4 °C, wells were washed 3 times with binding buffer and scanned directly on a Victor3 (PerkinElmer) or a ClarioStar (BMG Labtech) multiwell plate reader.

In all cases, averages of duplicate wells are plotted. In order to compare binding across the large concentration ranges tested, the fluorescence values at each concentration of each ligand were normalized to the maximum signal for that ligand and concentration. The values for the maximum signals for each ligand and concentration are indicated in Tables S1–S9. The corresponding expansion needed to make the maximum peak height for the more dilute concentrations equal to the maximum peak height at the highest concentration on the graph is indicated next to each concentration. For each ligand used to screen the array, the average percentage errors given in the legends were determined as the difference between the values for duplicate wells as a percentage of the average of the values (Benjamin et al. 2024). The overall average errors for each ligand were based on signals that were greater than 10% of the maximum signal. These values are provided in both the figure legends and the tables.

## Supplementary Material

Tables_S1-S9_revised_cwaf011

Supporting_information_cover_page_revised_cwaf011

## Data Availability

All data are contained in the manuscript and supporting information.

## CRediT statement

Stefi V Benjamin (Data curation [equal], Investigation [equal], Visualization [equal]), Maureen E Taylor (Conceptualization [equal], Funding acquisition [equal], Investigation [equal], Project administration [equal], Writing—original draft [equal], Writing—review & editing [equal]), Kurt Drickamer (Conceptualization [equal], Data curation [equal], Funding acquisition [equal], Investigation [equal], Project administration [equal], Writing—original draft [equal], Writing—review & editing [equal], Visualization [equal]).

## References

[ref1] Affandi AJ et al. 2020. Selective tumor antigen vaccine delivery to human CD169+ antigen-presenting cells using ganglioside-liposomes. Proc Natl Acad Sci USA. 117:27528–27539. 10.1073/pnas.2006186117. 33067394 PMC7959579

[ref2] Ahn G et al. 2021. LYTACs that engage the asialoglycoprotein receptor for targeted protein degradation. Nat Chem Biol. 17:937–946. 10.1038/s41589-021-00770-1. 33767387 PMC8387313

[ref3] Arena A et al. 2022. Improvement of lipoplexes with a sialic acid mimetic to target the C1858T PTPN22 variant for immunotherapy in endocrine autoimmunity. Front Immunol. 13:838331. 10.3389/fimmu.2022.838331. 35355982 PMC8959661

[ref4] Banik SM et al. 2020. Lysosome-targeting chimaeras for degradation of extracellular proteins. Nature. 584:291–297. 10.1038/s41586-020-2545-9. 32728216 PMC7727926

[ref5] Benjamin SV et al. 2024. A human lectin array for characterizing host-pathogen interactions. J Biol Chem. 300:107869. 10.1016/j.jbc.2024.107869. 39384043 PMC11566865

[ref6] Brannagan TH 3rd et al. 2022. Liver-directed drugs for transthyretin-mediated amyloidosis. J Peripher Nerv Syst. 27:228–237. 10.1111/jns.12519. 36345805 PMC10100204

[ref7] Coombs PJ, Graham SA, Drickamer K, Taylor ME. 2005. Selective binding of the scavenger receptor C-type lectin to Lewis^x^ trisaccharide and related glycan ligands. J Biol Chem. 280:22993–22999. 10.1074/jbc.M504197200. 15845541

[ref8] Dzionek A et al. 2001. BDCA-2, a novel plasmacytoid dendritic cell-specific type II C-type lectin, mediates antigen capture and is a potent inhibitor of interferon alpha/beta induction. J Exp Med. 194:1823–1834. 10.1084/jem.194.12.1823. 11748283 PMC2193584

[ref9] Feinberg H et al. 2017. Mechanism of pathogen recognition by human dectin-2. J Biol Chem. 292:13402–13414. 10.1074/jbc.M117.799080. 28652405 PMC5555199

[ref10] Fiete DJ, Beranek MC, Baenziger JU. 1998. A cysteine-rich domain of the ``mannose'' receptor mediates GalNAc-4-SO_4_ binding. Proc Natl Acad Sci USA. 95:2089–2093. 10.1073/pnas.95.5.2089. 9482843 PMC19259

[ref11] Gonzalez-Gil A, Schnaar RL. 2021. Siglec ligands. Cells. 10:1260. 10.3390/cells10051260. 34065256 PMC8161119

[ref12] Grabowski GA et al. 1995. Enzyme therapy in type 1 Gaucher disease: comparative efficacy of mannose-terminated glucocerebrosidase from natural and recombinant sources. Ann Intern Med. 122:33–39. 10.7326/0003-4819-122-1-199501010-00005. 7985893

[ref13] Graham SA et al. 2011. Identification of neutrophil granule glycoproteins as Lewis^x^-containing ligands cleared by the scavenger receptor C-type lectin. J Biol Chem. 286:24336–24349. 10.1074/jbc.M111.244772. 21561871 PMC3129213

[ref14] Huflejt ME, Leffler H. 2004. Galectin-4 in normal tissues and cancer. Glycoconj J. 20:247–255. 10.1023/B:GLYC.0000025819.54723.a0. 15115909

[ref15] Jégouzo SAF et al. 2015. A novel mechanism for binding of galactose-terminated glycans by the C-type carbohydrate recognition domain in blood dendritic cell antigen 2. J Biol Chem. 290:16759–16771. 10.1074/jbc.M115.660613. 25995448 PMC4505424

[ref16] Jégouzo SAF et al. 2020. Mammalian lectin arrays for screening host–microbe interactions. J Biol Chem. 295:4541–4555. 10.1074/jbc.RA120.012783. 32094229 PMC7135977

[ref17] Lee K et al. 2003. A biochemical and pharmacological comparison of enzyme replacement therapies for the glycolipid storage disorder Fabry disease. Glycobiology. 13:305–313. 10.1093/glycob/cwg034. 12626384

[ref18] Lin PM, Wright JR. 2006. Surfactant protein a binds to IgG and enhances phagocytosis of IgG-opsonized erythrocytes. Am J Physiol Lung Cell Mol Physiol. 291:L1199–L1206. 10.1152/ajplung.00188.2006. 17090701

[ref19] Liu W et al. 2004. Characterization of a novel C-type lectin-like gene, LSECtin: demonstration of carbohydrate binding and expression in sinusoidal endothelial cells of liver and lymph node. J Biol Chem. 279:18748–18758. 10.1074/jbc.M311227200. 14711836

[ref20] Liu L, Prudden AR, Bosman GP, Boons G-J. 2017. Improved isolation and characterization procedure of sialylglycopeptide from egg yolk powder. Carbohydr Res. 452:122–128. 10.1016/j.carres.2017.10.001. 29096185 PMC5705003

[ref21] Liu Y et al. 2024. Interactions that define the arrangement of sugar-binding sites in BDCA-2 and dectin-2 dimers. Glycobiology. 34:cwae082. 10.1093/glycob/cwae082. 39361900 PMC11632364

[ref22] McCormack FX, Stewart J, Voelker DR, Damodarasamy M. 1997. Alanine mutagenesis of surfactant protein a reveals that lipid binding and pH-dependent liposome aggregation are mediated by the carbohydrate recognition domain. Biochemistry. 36:13963–13971. 10.1021/bi970745q. 9374876

[ref23] Nair JK et al. 2014. Multivalent N-acetylgalactosamine-conjugated siRNA localizes in hepatocytes and elicits robust RNAi-mediated gene silencing. J Am Chem Soc. 136:16958–16961. 10.1021/ja505986a. 25434769

[ref24] Nonnecke EB et al. 2022. Human intelectin-2 (ITLN2) is selectively expressed by secretory Paneth cells. FASEB J. 36:e22200. 10.1096/fj.202101870R. 35182405 PMC9262044

[ref25] Nycholat CM et al. 2019. A sulfonamide sialoside analogue for targeting siglec-8 and -F on immune cells. J Am Chem Soc. 141:14032–14037. 10.1021/jacs.9b05769. 31460762 PMC6861165

[ref26] Ohtani K et al. 2001. The membrane-type collectin CL-P1 is a scavenger receptor on vascular endothelial cells. J Biol Chem. 276:44222–44228. 10.1074/jbc.M103942200. 11564734

[ref27] Powlesland AS et al. 2008. A novel mechanism for LSECtin binding to Ebola virus surface glycoprotein through truncated glycans. J Biol Chem. 283:593–602. 10.1074/jbc.M706292200. 17984090 PMC2275798

[ref28] Prakash TP et al. 2016. Comprehensive structure−activity relationship of triantennary n-acetylgalactosamine conjugated antisense oligonucleotides for targeted delivery to hepatocytes. J Med Chem. 59:2718–2733. 10.1021/acs.jmedchem.5b01948. 26914862

[ref29] Schlesinger PH et al. 1980. The role of extra-hepatic tissues in the receptor-mediated plasma clearance of glycoproteins terminated by mannose or *N*-acetylglucosamine. Biochem J. 192:597–606. 10.1042/bj1920597. 7236228 PMC1162375

[ref30] Shen W et al. 2024. Targeted delivery of herpes simplex virus glycoprotein D to CD169+ macrophages using ganglioside liposomes alleviates herpes simplex keratitis in mice. J Control Release. 365:208–218. 10.1016/j.jconrel.2023.11.026. 37981051

[ref31] Valverde P, Martínez JD, Cañada FJ, Ardá A, Jiménez-Barbero J. 2020. Molecular recognition in C-type lectins: the cases of DC-SIGN, langerin, MGL, and L-sectin. Chembiochem. 21:2999–3025. 10.1002/cbic.202000238. 32426893 PMC7276794

[ref33] Wesener DA et al. 2015. Recognition of microbial glycans by human intelectin-1. Nat Struct Mol Biol. 22:603–610. 10.1038/nsmb.3053. 26148048 PMC4526365

[ref34] Yamashita K, Koide N, Endo T, Iwaki Y, Kobata A. 1989. Altered glycosylation of serum transferrin of patients with hepatocellular carcinoma. J Biol Chem. 264:2415–2423. 10.1016/S0021-9258(19)81629-0. 2536709

