# Supplementary material for: Application of a human lectin array to rapid in vitro screening of sugar-based epitopes that can be used as targeting tags for therapeutics
Source: Glycobiology. 2025 Mar 2;35(4):cwaf011. doi: 10.1093/glycob/cwaf011 (PMC11903255; doi:10.1093/glycob/cwaf011)
Supplement: Supporting_information_cover_page_revised_cwaf011 [file supporting_information_cover_page_revised_cwaf011.pdf]

Supporting Information

Application of a human lectin array to rapid *in vitro* screening of sugar-based epitopes that can be used as targeting tags for therapeutics

Stefi V. Benjamin, Maureen E. Taylor, and Kurt Drickamer

Tables S1-S9. Binding data for Figures 2-9 [Excel file].
